# Calibrating well-being, quality of life and common mental disorder items: psychometric epidemiology in public mental health research

**DOI:** 10.1192/bjp.bp.115.165530

**Published:** 2016-08

**Authors:** Jan R. Böhnke, Tim J. Croudace

**Affiliations:** **Jan R. Böhnke**, Dr. rer. nat., Department of Health Sciences and Hull York Medical School, Mental Health and Addiction Research Group, University of York, York; **Tim J. Croudace**, PhD, MSc, BSc(Hons), DipAppPsych, School of Nursing & Health Sciences, University of Dundee, Dundee, UK

## Abstract

**Background**

The assessment of ‘general health and well-being’ in public mental health research stimulates debates around relative merits of questionnaire instruments and their items. Little evidence regarding alignment or differential advantages of instruments or items has appeared to date.

**Aims**

Population-based psychometric study of items employed in public mental health narratives.

**Method**

Multidimensional item response theory was applied to General Health Questionnaire (GHQ-12), Warwick-Edinburgh Mental Well-being Scale (WEMWBS) and EQ-5D items (Health Survey for England, 2010–2012; *n* = 19 290).

**Results**

A bifactor model provided the best account of the data and showed that the GHQ-12 and WEMWBS items assess mainly the same construct. Only one item of the EQ-5D showed relevant overlap with this dimension (anxiety/depression). Findings were corroborated by comparisons with alternative models and cross-validation analyses.

**Conclusions**

The consequences of this lack of differentiation (GHQ-12 *v.* WEMWBS) for mental health and well-being narratives deserves discussion to enrich debates on priorities in public mental health and its assessment.

Psychological assessment of populations in the form of self-report questionnaires and patient-reported outcome measures play a key role in psychiatric research, clinical epidemiology and in public (mental) health. The ability of questionnaire instruments to reliably assess multiple aspects of health has been questioned by recent research,^1–4^ and the finding that one dominant dimension explains a large proportion of the variability in many instruments has consequences for debates about the (psychometric) epidemiology of population health and well-being.^5,6^ How do we accurately assess the psychological well-being of a nation, be it once or repeatedly? With which sets of instruments or items? Many governments are exploring strategies to assess population well-being using both established or experimental measures to inform or evaluate policy with some broader notions than economic measures of social progress.^7,8^ We report the first epidemiological study with contemporary psychometric techniques of the (dis)similarities of instruments that appear in many archived UK Government surveys and that are also discussed as future measures: Goldberg's General Health Questionnaire (GHQ) items^9^ and the more recently developed Warwick-Edinburgh Mental Well-being Scale (WEMWBS).^10^ This article reports on a psychometric evaluation of whether (or to which degree) the two instruments assess different constructs in representative UK population samples. The analysis also includes the items of the EQ-5D^11^ to corroborate that the chosen analytic methods can detect differences between items that address related, but sufficiently different aspects of subjective health.

## Method

### Participants

We studied respondents from three waves of the Health Survey for England (HSE, 2010,^12^ 2011,^13^ 2012^14^). Our analyses included respondents older than 16 who were scheduled for the survey's self-completion module with the three relevant instruments. Since the HSE is run on a household basis, we selected up to two respondents from the same household (one for the estimation sample and a second, different one for the validation sample^15^). Table 1 provides information on the number of such respondents available for analysis.

**Table 1:** Number of respondents in each wave of the Health Survey for England (HSE) and number of respondents that had at least one response on the respective instrument

	Total respondents			Respondents with at least one response on:		
	*n*	Women, %	Age, years: mean (s.d.)	EQ-5D, *n*	12-item General Health Questionnaire	Warwick-Edinburgh Mental Well-being Scale
HSE 2010	7255	56.9	50.52 (18.51)	7234	7223	7153

HSE 2011	7246	56.5	49.91 (18.25)	7182	?	7163

HSE 2012	4789	56.7	52.32 (17.91)	4742	4739	4779

For efficiency, the survey responses were pooled across all three data-sets (total *n* = 19 290). The use of partly incomplete data (since the 12-item GHQ (GHQ-12) was not collected in 2011) is an instance of a ‘planned missing data’ design for which standard solutions from the analytical literature on integrative data analysis exist.^16–18^ Random sample splits generated one estimation sample (*n* = 9669) – in which the statistical models were optimised – and a validation sample (*n* = 9621) that we evaluated to test whether our best-fitting model was not purely a result of overfitting to one data-set, a possibility we hoped to avoid.^19^

### Measures

The GHQ^9^ is a self-report questionnaire aimed at screening for diagnosable levels of psychological distress in healthcare settings. The GHQ-12 has been shown to provide excellent reliability and validity as a screen for risk of common mental disorder. It is popular because of its brevity and its frequent use provides comparability across studies.^20^ An important feature of the GHQ-12 is its inclusion of six positively and six negatively phrased items that have different verbal descriptors of their response options. Items are laid-out so that higher and right-most responses indicate greater distress. Whether these items measure one distress dimension and a method factor (correcting for wording effects) or two correlated dimensions is a topic of debate^15,21–23^ and will be addressed by our statistical analysis (see below and online supplement DS1).

The WEMWBS aims at assessing mental well-being and its emotional, cognitive and psychological aspects.^10^ The WEMWBS was designed to provide a short (14 items) and reliable assessment with solely positively phrased items. All items have five response options, anchored from ‘None of the time’ to ‘All of the time’. Higher scores indicate ‘well-being’. Studies have reported it to be a reliable instrument,^24^ largely unidimensional,^25^ with possible value in predicting health-related behaviours.^26^

We included the EQ-5D (EuroQol)^11,27^ as a credible set of items to gauge the sensitivity of our analytic strategy to the presence of discriminant validity. The five items each assess a dimension of health-related quality of life (mobility, self-care, usual activities, pain/discomfort and anxiety/depression). Responses to these items are captured with three response options and item-specific anchors (for example, mobility: ‘no problems walking about’, ‘some problems walking about’, ‘confined to bed’). Although researchers increasingly use the score of the EQ-5D as a dimensional indicator of health-related quality of life, it is important to note that the items were not developed as indicators of a latent dimension and are used in this study solely to explore discriminant validity. Instead, the EQ-5D was developed and validated to order 243 different health states, i.e. response patterns, according to their relative valuations in population samples.^11^

### Statistical analysis

Item response factor models can be used (a) to establish the number of latent variables needed to explain the responses to the *k* = 31 items of the three instruments and (b) to investigate relationships between latent variables.^28^ All models were estimated with Mplus 7.11^29^ using full information maximum likelihood (FIML) estimation.^30^ To explore the dimensional structure of the questionnaires^4,31^ we assessed first the fit of a one-dimensional model (model 1) to test whether the instruments all measure the same latent variable (for example, well-being). We then assessed an exploratory correlated factor model with three factors (model 2) to test whether the instruments are separable and therefore assess different correlated constructs. The last set of models (model 3 and model 4) were bifactor models.^32^ Bifactor models assume that all items load on one general dimension, but that there are remaining sources of covariation because of common characteristics of certain item sets. Model fit was assessed based on information criteria that identify the best description of the data while penalising for model complexity.^33^ The best-fitting model was cross-validated in the validation sample.

To evaluate the relative importance of the identified dimensions, we first calculated (partial) test information functions, i.e. the inverse of the measurement error. These functions provide a graphical evaluation of how accurately different sets of items assess the latent factor (relative efficiency).^34,35^ The second strategy aimed at assessing the amount of score variance as a result of each factor,^32^ i.e. assessing the reliability of the general factor and the three instrument-specific factors. To this end, we evaluated omega (ω) coefficients. Of these, ω (without subscript) assesses the share of score variance as a result of all factors of the bifactor model taken together (general, specific, method); and omega-h (ω_h_) in turn only assesses the share of score variance as a result of the general factor, i.e. the reliability of the general factor alone. We also assessed ω coefficients for the three instruments (GHQ-12, WEMWBS, EQ-5D) individually, which provide the reliability of each instrument as derived from the bifactor model, as well as omgea-s (ω_s_) coefficients, which assess the specific measurement quality of each instrument, when the general factor is partialled out.^36^

## Results

For comprehensiveness, factor analyses for individual instruments appear in online supplement DS1. These reveal that one dimension explained most of the covariance in responses to both the GHQ-12 and the WEMWBS' 14 items. The model fit for each instrument was further optimised by adding a so-called ‘method factor’ for the negatively phrased items of the GHQ-12 and a factor for items dealing with social contacts and interests in the WEMWBS. These factors were retained in the following analyses. A confirmatory bifactor model with specific factors for the three instruments as well as two factors for wording effects (GHQ-12 items) and social/interest items (WEMWBS; model 4) was the best description of the data while penalising for complexity (Table 2) as indicated by both Bayesian information criterion (BIC) and adjusted BIC (BIC_adj_, lowest values of all estimated models^33^). We discuss the results of three models in more detail: unidimensional, exploratory and bifactor.

**Table 2:** Information criteria for the factor models with all items across the three instruments (12-item General Health Questionnaire, Warwick-Edinburgh Mental Well-being Scale and EQ-5D) in the estimation sample^a^

	Model 1: confirmatory one factor	Model 2: exploratory three factor, geomin-rotated	Model 3: confirmatory bifactor^b^	Model 4: confirmatory bifactor with two method factors^c^
Log likelihood (*LL*)	−209 811	−198 980	−199 699	−198 689

Number of parameters (*n*(P))	133	192	164	175

Bayesian information criterion (BIC)	420 843	399 722	400 903	398 984

Adjusted BIC	420 420	399 111	400 381	398 428

a. *n* = 9661; estimator: full information maximum likelihood.

b. This model estimates only the general factor and three specific factors for the three instruments and was only estimated to test whether the addition of the method factors was necessary in model 4; since model 4 showed better fit, this model is not further discussed.

c. See Fig. 1 for details.

### Unidimensional

The unidimensional model (model 1; Table 2) assumes that one latent factor causes the responses to all three instruments. The items of both GHQ-12 and WEMWBS show high loadings on this factor (the lowest loading was *r* = 0.54; online Table DS1). Based on a minimal loading^37^ of *r* = 0.40 three of the EQ-5D items load relevantly on this factor: anxiety/depression (*r* = 0.71), self-care (*r* = 0.54) and usual activities (*r* = 0.47). Compared with the mean loading of 0.68 (s.d. = 0.10) of the GHQ-12 and WEMWBS items on this general factor, the latter two items load only marginally on this factor.

### Exploratory

The exploratory model (model 2; Table 2) investigates whether three correlated latent constructs are present. The resulting factors are moderately correlated (see footnote to online Table DS1) and the first factor is highly loaded by items from the EQ-5D but only moderately so by some items from the other instruments. The second and third factors mirror the structure of the GHQ-12 and WEMWBS, with the anxiety/depression item of the EQ-5D loading with the GHQ-12 items (on the second factor).

The results from both the unidimensional and exploratory models indicate that instruments developed for very different purposes (GHQ-12 for screening; WEMWBS for mental well-being) share a considerable amount of variance and overlap in their measured range of distress or well-being.

### Bifactor

The bifactor model (Fig. 1; model 4, Table 2) includes a (general) factor for all items, three (specific) factors for each instrument and also the two method factors that were identified in the single scale analyses (see online supplement DS1). The GHQ-12 items show equal or higher loadings on the general factor than expected from the results of the earlier unidimensional model (online Table DS1). The fact that no item shows higher loadings on the specific factors for negative wording or the scale itself indicates that the general factor is a reliable description of the response process for these items: the GHQ-12 items in effect define the metric of this (general/dominant) factor and variations along this continuum are anchored by their item content.

**Fig. 1:**
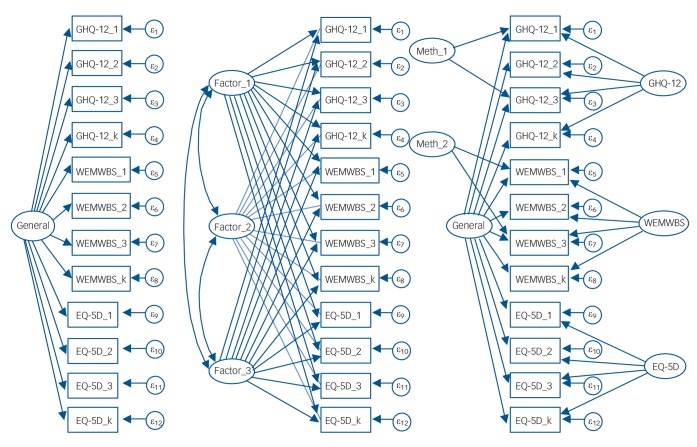
Schematic representation of models 1, 2, and 4 (from left to right). Arrows indicate loadings of an observed item (box) from the three instruments (12-item General Health Questionnaire (GHQ-12), Warwick-Edinburgh Mental Well-being Scale (WEMWBS), EQ-5D) on a latent variable (circle); Meth_1 indicates the method factor for wording of the GHQ-12 items; Meth_2 indicates the method factor for interest/social items of the WEMWBS items; model 3 is equivalent to model 4 without either of these method factors.

The items of the WEMWBS show lower loadings on this general factor than expected from the unidimensional solution and its left-over covariation is captured both by the specific factor for the instrument and the factor for the social/interest items. Both of these specific factors show higher loadings for most items than on the general factor, i.e. these items tap into aspects beyond the general factor (i.e. detectable multidimensionality beyond the established GHQ-12 metric).

The anxiety/depression item of the EQ-5D aligns with the GHQ-12 metric, again ‘standing off’ from the other four items. After extraction of the general factor, the self-care and usual-activities items align with the other two non-mental-health items.

### Cross-validation

To examine the robustness of our results, we treated the parameters (loadings and category thresholds) from the estimation sample as fixed and assessed how well they fitted the data of our validation sample.^4,19^ Model 4 described the sample better than the closest runner-up, model 3 without the method factors (Table 3). Both models were re-estimated with weighted least squares means and variance adjusted (WLSMV) in the estimation sample.^38^ The results were structurally very similar to the FIML solution and they were also checked in the validation sample. This procedure allowed for the estimation of typical fit indices for structural equation models that indicated good fit (Table 3) and also a model comparison test between the two solutions could be estimated that was highly significant (χ^2^ = 1123.03, d.f. = 2, *P*<0.001). All indications are therefore supportive of the modelling claims and support the results found in the original analysis.

**Table 3:** Model fit for cross-validation in the validation sample

	All parameters fixed		Free variances	
	Model 3:^b^ bifactor	Model 4: full bifactor	Model 3:^b^ bifactor	Model 4: full bifactor
Log likelihood (*LL*)	−198 233	−197 031	−198 233	−197 010

Number of parameters (*n*(P))	0	0	4	6

Bayesian information criterion (BIC)	396 468	394 061	396 502	394 074

Adjusted BIC	396 468	394 061	396 490	394 055

Root mean square error of approximation (90% CI)^c^	–	–	0.035 (0.034-0.035)	0.032 (0.031-0.033)

Tucker-Lewis index/comparative fit index^c^	–	–	0.98/0.98	0.98/0.99

a. *n* = 9614–9621 not completely missing responses.

b. This model estimates only the general factor and three specific factors for the three instruments and was only estimated to test whether the addition of the method factors was necessary in model 4.

c. Available only for model estimates from weighted least squares means and variance adjusted estimation (WLSMV) with *n*(P)>1.

### Information across the measurement range

**Fig. 2:**
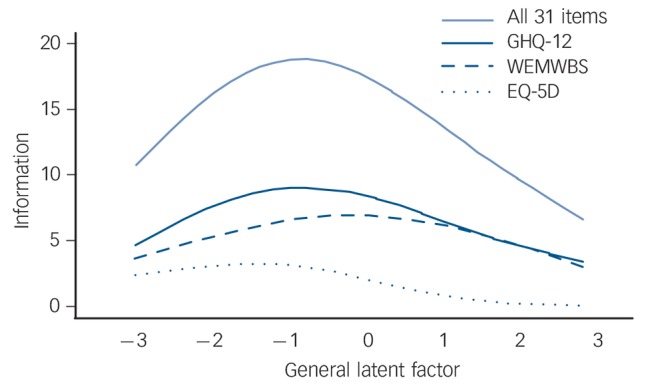
Test information function for all 31 items of the three instruments (pale blue solid line) and partial test information functions for the items of the 12-item General Health Questionnaire (GHQ-12, dark blue solid line), the Warwick-Edinburgh Mental Well-being Scale (WEMWBS, dark blue dashed line) and the EQ-5D (dark blue dotted line). The latent trait is the general dimension from the full bifactor model. The latent trait values are standardised with s.d. = 1 and ‘0’ indicates the overall population mean on the general factor.

All items (except four items of the EQ-5D) assess one strong dimension, making it of interest to understand how well the different items span the psychometric measurement range of this new factor. Item response theory-type bifactor analyses allow us to assess the range of the latent factor on which the item sets provide information as well as their relative contribution (relative efficiency^34,35^). The so-called information function (Fig. 2)^39^ shows the inverse of the measurement error at each estimated latent score value (*x*-axis), hence more information indicates higher measurement precision for a given score level. Multiple lines offer direct comparisons of scales, with the pale blue line illustrating the information provided by the *k* = 31 items of the three instruments together. This information function has its peak between the sample mean (at ‘0’ in Fig. 2) and a value almost two standard deviations below it. This is the region on which all items together provide most of the information and the standard error of measurement is the smallest. Given that this is a general population sample, the items on the whole span levels of (mild) distress.

The dark blue lines present how each instrument contributes separately to the measurement of the general factor (‘partial information function’). The GHQ-12 has a higher information function (solid dark blue line) than the WEMWBS (dark blue dashed line) up to one standard deviation above the population mean. Beyond that, both instruments provide about the same amount of information (have similar accuracy). Since only one item of the EQ-5D (dark blue dotted line) loads relevantly on the general factor, it contributes only little to the measurement accuracy compared with the other 26 items.

### Reliabilities

The ω = 0.98 (online Table DS1) indicated that nearly all of the variance observed across the scores of the *k* = 31 items could be attributed to the six sources that we defined in the bifactor model (i.e. the latent factors depicted in Fig. 1). The ω_h_ = 0.81 for the general factor alone indicates still a reasonable reliability for the joint dimension across all items, but the difference between these two numbers shows that a substantial proportion of the score variance was as a result of the five specific factors of the model. Although the ω = 0.96 across all GHQ-12 items indicates a high reliability, extracting the variance in GHQ-12 scores as a result of the general factor reduces this to an ω_s_ = 0.11 – the GHQ-12 items contain no specific variance over and above the general factor. For the WEMWBS (ω = 0.95; ω_s_ = 0.44) and especially the EQ-5D (ω = 0.94; ω_s_ = 0.61) the results are less dramatic: both assess at least to some degree a specific dimension.

## Discussion

To our knowledge this is the first study to comprehensively test the dimensionality of three instruments used to inform public mental health policy in the general population that also quantifies how differently they function in such a sample using modern psychometric methods.^6^ Three general population samples and an established approach to merge multiple data sources were applied and the results showed that the GHQ-12 and the WEMWBS share one dimension and their items rather assess health states below the general population mean than above. The latter would be required to indicate a focus on positive states, and to potentially extend the continuum into ‘well-being’. The finding of a strong general factor converges with other findings^3,4,31,39–42^ and underlines the necessity to test whether alternatively developed and variously titled measures actually provide independent information.^1,6,43^ It is not possible to conclude this from instrument/item labelling or correlations between instrument scores alone, although that is often argued.

The bifactor modelling approach that we applied is a particularly powerful test for multidimensionality. For two instruments to assess different constructs the loadings on the general factor should be low at least for one instrument and the corresponding specific factor should show relevant loadings for all items of that scale. Both the GHQ-12 and the WEMWBS fail this test and for both instruments more than 50% of the common variance is because of the general dimension.^32^ Although this finding questions the differential validity of the two instruments, on a more positive note it shows that the item responses from both instruments can be translated into each other since they are strongly connected by the general dimension.^18,44^ Our results show that this is not advisable for the EQ-5D: it shares only a comparatively small amount of variation with the items of the other two instruments. This result also shows that the applied approach can identify differences between items that might be used in a similar setting, but that are geared at assessing different aspects of health and well-being. Therefore, the overlap between the GHQ-12, the WEMWBS and the single item of the EQ-5D is unlikely to be (only) a consequence of the statistical procedure.

### Implications for well-being research and public mental health

It has been argued that a deficit-oriented perspective on the distress derived from symptoms of mental disorders should be complemented by a positive perspective emphasising ‘well-being’,^7,45,46^ which has been a central feature in the narratives offered by proponents of positive psychology.^47^ It appears closer to the broad definition of ‘health’ offered and endorsed by the World Health Organization.^48^ Scholars working from the evidence base on mental disorders and psychiatric distress have argued that in populations shifting (mental) well-being to be more positive would decrease the prevalence and incidence of psychopathological symptoms or even disorder.^6,49^ And last but not least, many governments consider well-being of their populations as a core assessment beyond gross domestic product, as a wider measure of economic and social progress.^7,8^ One recent (but perhaps not definitive) consultation on this topic in the UK ended with the WEMWBS being favoured over the GHQ-12: the ‘GHQ-12 is a well validated and well used measure of mental health, however it is a screening instrument of mental illness, not a measure of mental wellbeing’.^50^

Whether two instruments developed for different purposes actually measure different constructs, is an empirical question.^1,43^ Our research highlights that the latent constructs assessed by two instruments overlap to a far greater extent than expected from the consultation statement. It was well known that responses to these two instruments are correlated,^7,24,51^ but our study shows compellingly that in the UK population all items of the two instruments share a common dimension, which explains a considerable share of their score variance.

The most important test for superiority of one instrument above the other is still missing: to establish whether one of the instruments is actually a better predictor of an agreed gold-standard of what ‘well-being’ really is. This remains a difficult challenge, but one not unique to well-being research in public mental health. It also applies to many social science concepts and often to ‘caseness’ in psychiatry. In our proposed framework the general factor being more highly correlated with the criterion would be conclusive evidence that the common part across items is closest to the criterion (for example, ‘well-being’). If instead one of the specific latent scores is actually a better predictor than the other factor scores and/or the general factor, this would be evidence that one instrument is a more useful representation of the construct in question than the other.

We feel that there is still a large gap in the existing knowledge base and perhaps lack of consensus about (a) the relative independence of the variety of instruments to measure well-being, (b) the relative predictive power for any target or gold-standard reference that should be assessed and (c) the nature of this gold-standard criterion. If self-report assessments shall take on the task of assessing well-being in the population to guide and evaluate the effects of policy, we currently see a pressing need for research regarding all these aspects.^6^ Integrating our proposed framework into standard investigations of the predictive value of a collection of items (for example, health risks or behaviours^26,52^) might help to close this gap.

If well-being is in fact a multidimensional construct,^7,46,53^ the finding of a strong general factor in a small set of items is not surprising. Best psychometric practice would construe a scale for every aspect of well-being.^54^ Aggregating across a range of different (sub)constructs of well-being (for example, mastery, personal relationships, hedonic aspects^7^) by building a single scale (i.e. a single score) across heterogeneous indicators may lead to the situation that only an evaluation of ‘negativity’ is the underlying commonality: most respondents can agree when they are lacking well-being, but the positive end-points might then differ across individuals to the extent that a unidimensional assessment of these is difficult to obtain.^55^

### Strengths and limitations

The use of three UK-representative survey data-sets allowed a robust assessment of the research question. The large combined size enabled us to split the sample and to test the structure on a statistically independent sample. The use of current psychometric methodology enabled a detailed assessment of the items, the instruments and their scores. And although the study contained some exploratory elements, it was largely a confirmatory test of the differences between instruments that we might expect to appear in the evidence base to guide implementation of well-being assessments.

The greatest shortcoming is the lack of an external reference criterion of well-being to test its differential association with the GHQ-12 and the WEMWBS. A second limitation addresses the meaning of the general factor: although it is convenient to assume that it captures the common substantive variation across instruments, it instead could capture mainly methodological artefacts across the three instruments.^56^ To clarify, we note that we were not able to test this in the current data-sets and so it remains another pressing research hypothesis for the use of self-report questionnaire data in general health surveys.^5^ A third limitation is that we deliberately did not give new or preferred definitions of what well-being is. We used common instruments that have been discussed as indicators or measures of public mental health.^7,50^ The lack of finding major difference between measures can be for several reasons: (a) the respondents not being able to differentiate, (b) the instruments actually assessing one factor, or (c) a lack of theoretical differentiation when constructing the questionnaires. Fourth, we used what we consider ‘short’ instruments – those already deployed in surveys because of their brevity. We note that these are considerably shorter than they were at prior stages in their development and evaluation phases^9,10,57^ and analyses of the long versions might come to different conclusions with regard to the importance of each of our factors.

Finally, we focused only on the aspect of similarity/dissimilarity of these measures. We did not consider other important aspects relevant to the assessment of population well-being such as the fairness of these instruments across regions or demographic groups in the UK, which would be needed to provide assessments that can reflect the state of well-being in a vibrant and fair society.^5^ We think that this would distract from the primary message of the research we report here, which is that a much more nuanced discussion about well-being as an epidemiological dimension has to be held. The next step would then be to ensure that the chosen item collection allows a fair assessment.

In conclusion, contemporary psychometric methods and data integration offer a promising direction for future research innovations. Inherent in this perspective is our discouragement to other researchers to think only about testing or deploying existing single instruments, instead shifting the focus to testing ‘collections of items’: (a) to identify items that enable an optimal comparison on one general dimension (the general factor in our application), (b) to cover as many domain aspects as necessary in public and academic opinion (specific factors in this case) as well as (c) selecting those items that are maximally predictive of an external gold-standard of well-being. Collections of items and scales should be used, since there are no good scales but only good items for specific purposes.^5,58^ And for assessing and improving the public's mental health and well-being the best items available should be chosen. Applying the proposed framework, items would also become linkable with different instruments across samples and studies.^2,59^ This would be a truly contemporary psychometric epidemiological perspective on the well-being of populations.
